# Establishing neural representations for new word forms in 12-month-old infants

**DOI:** 10.3389/fnhum.2024.1386207

**Published:** 2024-06-13

**Authors:** Sari Ylinen, Emma Suppanen, István Winkler, Teija Kujala

**Affiliations:** ^1^Logopedics, Welfare Sciences, Faculty of Social Sciences, Tampere University, Tampere, Finland; ^2^Cognitive Brain Research Unit, Centre of Excellence in Music, Mind, Body and Brain, Department of Psychology and Logopedics, Faculty of Medicine, University of Helsinki, Helsinki, Finland; ^3^Institute of Cognitive Neuroscience and Psychology, HUN-REN Research Centre for Natural Sciences, Budapest, Hungary

**Keywords:** auditory processing, electroencephalography (EEG), event-related potential (ERP), language development, word learning

## Abstract

During the first year of life, infants start to learn the lexicon of their native language. Word learning includes the establishment of longer-term representations for the phonological form and the meaning of the word in the brain, as well as the link between them. However, it is not known how the brain processes word forms immediately after they have been learned. We familiarized 12-month-old infants (N = 52) with two pseudowords and studied their neural signatures. Specifically, we determined whether a newly learned word form elicits neural signatures similar to those observed when a known word is recognized (i.e., when a well-established word representation is activated, eliciting enhanced mismatch responses) or whether the processing of a newly learned word form shows the suppression of the neural response along with the principles of predictive coding of a learned rule (i.e., the order of the syllables of the new word form). The pattern of results obtained in the current study suggests that recognized word forms elicit a mismatch response of negative polarity, similar to newly learned and previously known words with an established representation in long-term memory. In contrast, prediction errors caused by acoustic novelty or deviation from the expected order in a sequence of (pseudo)words elicit responses of positive polarity. This suggests that electric brain activity is not fully explained by the predictive coding framework.

## Introduction

Infants readily learn from their auditory environment, including features of their native language spoken by their family. For example, infants can extract different patterns or rules from speech they hear (statistical learning; Gómez and Gerken, 2000; Saffran and Kirkham, 2018) and make predictions based on them (Emberson et al., 2019; Suppanen et al., 2022). Our previous research has suggested that the ability of newborn infants to learn from speech exposure and to make predictions about future input is linked to their later language skills (Suppanen et al., 2022). Learning from speech also enables infants to start building their mental lexicon during the first year of life, which requires the establishment of word representations in the brain that link the phonological representation of the word form with the corresponding semantic representation (Gupta and Tisdale, 2009). These neural representations serve as top-down templates and enable infants to recognize words from bottom-up input and understand their meaning. These neural representations are also reflected in infant brain activity: previous studies have shown distinct brain responses for learned words and unknown words or pseudowords in infants at 12–16 months (Molfese, 1989, 1990; Molfese et al., 1993; Mills et al., 1997, 2004; St. George and Mills, 2001; Ylinen et al., 2017).

Because newborn infants do not yet have long-term representations of words, their learning from speech input may rather be dominated by learning regularities, patterns, or rules and using them in predictive processing. According to the predictive coding theory, during perception, feedback signals are generated in a hierarchically organized neural network to predict the perceptual input. The difference between the predicted and actual input drives changes in the predictions to minimize this difference, thereby reducing surprise (Rao and Ballard, 1999) or free energy (Friston, 2005). As a result, predicted items result in weak or no prediction error signals (i.e., weaker brain responses), whereas unpredicted items evoke strong prediction error signals (i.e., stronger brain responses). This pattern was observed in our previous study of newborn infants (Suppanen et al., 2022). However, it is not clear how the processes and neural signatures of prediction and recognition change when infants are able to establish word representations during the second half of the first year of life. Our previous study (Ylinen et al., 2017) utilized disyllabic words and a study paradigm in which generating predictions of word endings based on word beginnings resulted in either enhanced negative-polarity mismatch responses (MMRs) due to the activation of long-term representations for a familiar word, or prominent positive-polarity prediction error responses for an unfamiliar word form in 12-month-old infants. While these results concern the processing of previously learned words, they raise the question of how the infant brain processes newly learned word forms at the same age. To this end, here we studied whether, in 12-month-old infants, a newly learned word form elicits neural signatures that resemble those of the recognition of a word by activation of an established word representation (Ylinen et al., 2017), or, rather, the processing of a newly learned word form shows the suppression of the neural response along with the principles of predictive encoding of a learned rule.

To study the learning of novel word forms, we presented infants with pseudowords in an experiment comprising two phases: (1) a familiarization phase in which the infants were presented with two spoken native-language disyllabic pseudowords (designated as “AB” and “CD,” where A, B, C, and D denote different syllables), and (2) a test phase with an oddball sequence in which one of the familiarized pseudowords (AB) served as the frequent standard stimulus interspersed with three rare deviant word forms (pseudowords or actual words): CD, AD, and AX (where X represents a syllable that did not appear during familiarization). The AD deviant was an actual word that is often known by 12-month-old infants: ‘kukka’ (/kuk:a/; a flower). The first syllable of CD was expected to elicit a frontocentral positive-polarity MMR for the acoustic change from A to C [for reviews of the mismatch negativity (MMN) or MMRs in adults and infants, see Näätänen, 2001; Kujala et al., 2023]. In addition, since we were particularly interested in word-level processing, six hypotheses were tested regarding how infants process the second syllables of the (pseudo)words, for which processing cues commence at the onset of the second syllable (300 ms from word onset in the current study).

- Because the auditory sequence with the frequent stimulus AB was likely to create a prediction for the repetition of AB, the syllables D of AD, X of AX, and C of CD could all elicit MMRs, reflecting the prediction error within the sound sequence (Ylinen et al., 2017). These MMRs are expected to have a frontocentral scalp distribution, and they could be either negative or positive in their polarity (see Näätänen et al., 2019, for a review). At 6–12 months, their latency has been reported to range from approximately 150 ms (Ylinen et al., 2017) to 450 ms from change onset (Cheng et al., 2013), depending on the characteristics of the stimuli and their context. Therefore, in the majority of cases, it is difficult to set specific hypotheses about MMR latency (see Näätänen et al., 2019), but the responses of interest were expected to occur between 150 and 450 ms from the onset of the second syllable.
- The first syllables of the familiarized disyllabic word forms create predictions for their familiarized second syllable. Because AD and AX violate the familiarized continuation of the AB word form, they should cause within-word prediction errors, as shown in our previous studies (Ylinen et al., 2017; Suppanen et al., 2022). However, these prediction errors are expected to differ from each other (see additional hypotheses III and IV; Suppanen et al., 2022).
- The response to the syllable X in AX should show the effects of novelty, as the X syllable has not appeared during the familiarization phase, and it was also rare within the test sequences. Novelty is typically associated with a frontocentral positive-polarity auditory ERP component in both infants and adults (see Kushnerenko et al., 2013, for a review). In our previous study with neonates (Suppanen et al., 2022), stimulus AX elicited a robust positive response that peaked at 300 ms from change onset. At 12 months of age, the latency may be slightly shorter due to maturation.
- Because AD is an actual word that could have been learned by the infants in their normal language environment, AD may elicit an enhanced response representing word or word-form recognition (Pulvermüller et al., 2001; for the long-term memory contribution to the MMN, see also Näätänen et al., 1997; Winkler et al., 1999; Ylinen et al., 2010). Based on our previous study in the same age group delivering the same stimulus as in the current study (but with a different kind of context in the sound sequence; Ylinen et al., 2017), we expected the word recognition response to be of negative polarity.
- The response to D in the deviant CD might be suppressed if predicted based on the learned rule that C is followed by D.
- Alternatively, the response to D in the deviant CD might elicit an enhanced response of negative polarity, similar to what was hypothesized for a real word AD (hypothesis IV), if CD activates a word-form representation established during the learning phase. (Note that Hypotheses V and VI are mutually exclusive.)

## Methods

### Ethics statement

The study protocol was approved by the Ethics Committee for Gynecology and Obstetrics, Pediatrics, and Psychiatry of the Hospital District of Helsinki and Uusimaa, Finland. Participants’ parents gave their informed written consent.

### Participants

This study was part of a larger project (Suppanen et al., 2022) in which 75 healthy, full-term newborn infants born into Finnish-speaking families were studied (see Suppanen et al., 2022, for details). Of these 75 infants, 68 participated in an EEG measurement at 12 months of age. Data from 16 participants were excluded due to participants missing or discontinuing the EEG recording, technical problems, or failure to meet the criterion of 50 accepted epochs per stimulus type. Thus, the data from 52 participants were included in the analyses (26 boys and 26 girls, average age 369 days, and SD 14 days).

### Stimuli and study design

**Figure 1:**
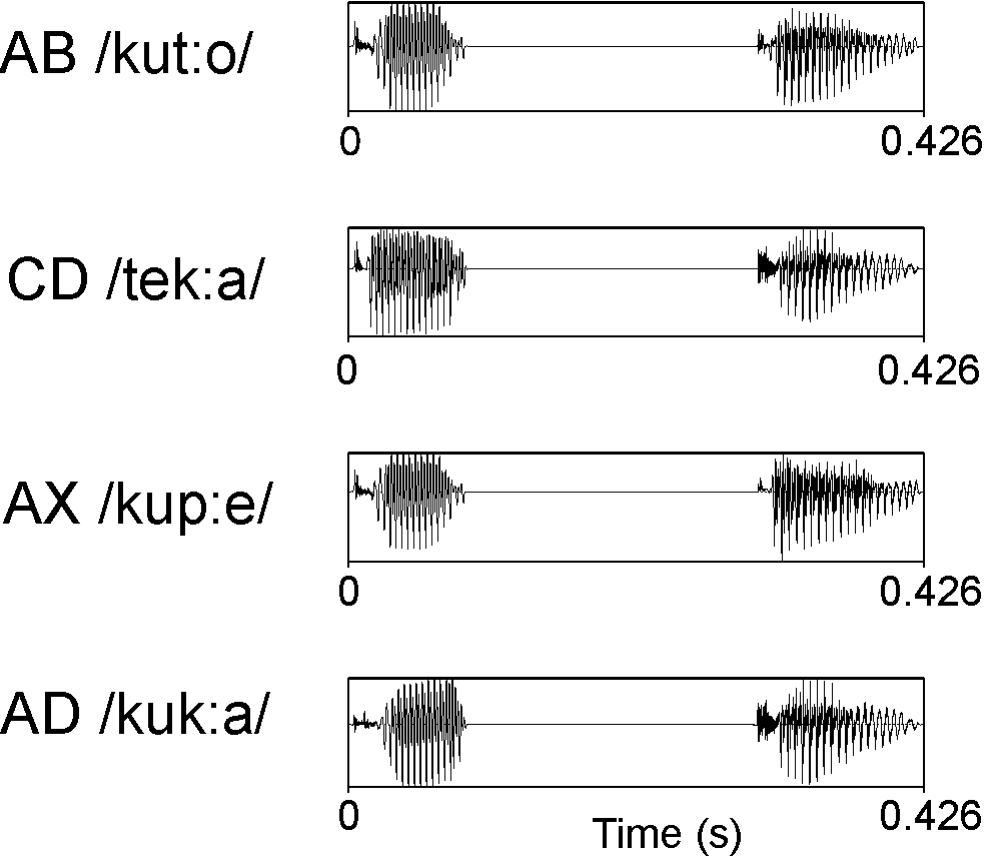
Waveforms of example stimuli spoken by a female native speaker of Finnish.

The auditory stimuli (see Figure 1) consisted of the phonotactically legal Finnish disyllabic pseudowords AB (/kut:o/), CD (/tek:a/), and AX (/kup:e/) and the word AD (/kuk:a/, which means flower). They were spoken in a sound-isolated studio by two native speakers of the Finnish language (one male and one female). For each syllable, the two most prototypical exemplars without clear co-articulatory cues revealing the original context were selected from each speaker and further processed with Praat (Boersma and Weenink, 2010). The intensities of the syllables in each position were matched as closely as possible. The duration of the stimuli was adjusted to 426 ms (the first syllable was 90 ms, a silent pause mimicking the occlusion phase of a stop consonant was 210 ms, and the second syllable was 126 ms). The onset of the second syllable was at 300 ms from the stimulus onset. Some variation in F0 was allowed within each speaker because we aimed for natural-sounding stimuli (for acoustic details, see Supplementary Table S1).

In the familiarization phase, the disyllabic pseudowords /kut:o/ and /tek:a/, denoted here as AB and CD, respectively, were presented to the participants with 50% probability (3 blocks, each having 250 stimuli; total duration 11.7 min). In the familiarization phase, half of the infants heard sequences in which 80% of the stimuli were spoken by a female speaker and the rest by a male speaker; the ratios were reversed in the other half of the infants.^1^ In the test phase, participants were presented with four oddball blocks (540 stimuli in each block, total duration 33.7 min) with the familiarized pseudoword AB as the standard stimulus (*p* = 0.79) and three other word forms (CD, AD, and AX) as deviants (*p* = 0.07 for each). The test phase took place immediately after the familiarization phase. The interstimulus interval (offset to onset) was 510 ms in both phases. The total recording time was approximately 45 min.

The presentation order was randomized with the following constraints: Each stimulus block started with at least eight standard stimuli, and at least two standards followed each deviant. Stimuli in the test phase were spoken by the same speaker, with half of the infants receiving male-only stimuli and the other receiving female-only stimuli in a counterbalanced fashion. All the data were pooled together for the current data analysis.

### Data acquisition and procedure

EEG data were recorded with 16 active electrodes placed according to the international 10–20 system (Fp1/2, F3/4, Fz, C3/4, Cz, P3/4, Pz, O1/2, Oz), with additional electrodes on the left and right mastoids (LM and RM). The used amplifier was QuickAmp (version 10.08.14; Brain Products GmbH, Gilching, Germany), and the recording software was BrainVision Recorder (version 1.20.0801; Brain Products GmbH). The sampling rate was 500 Hz with a 100 Hz online lowpass filtering cutoff frequency. The recording reference was the average of all electrodes.

The participants were awake and sitting on their parents’ laps during the measurement, and the parents entertained the participants during the measurement by silently showing them toys. The stimuli were presented in Presentation 17.2 Software (Neurobehavioral Systems Ltd., Berkeley, CA, United States) via two Genelec speakers: (Genelec Oy, Iisalmi, Finland) placed front left and front right approximately 160 cm from the participant. The approximate sound pressure level (SPL) was 65 dB.

### Data analysis

Only the data collected in the test phase are reported here. The data were preprocessed using BESA Research 6.0 (Besa GmbH, Gräfelting, Germany), MATLAB Release 2018b (The MathWorks Inc., Natick, Massachusetts, United States), EEGlab 2019.0 (Delorme and Makeig, 2004), and in-house MATLAB scripts (CBRUPlugin2.1b, Tommi Makkonen, Cognitive Brain Research Unit, University of Helsinki). The data were first bandpass-filtered offline (0.5–30 Hz, 24 dB/octave), re-referenced to the average of the two mastoid signals (RM and LM), and segmented into −100 to 800 ms epochs with respect to stimulus onset, separately for each stimulus and participant. The epochs were baseline-corrected by the average voltage in the 100 ms pre-stimulus interval. Epochs with an absolute amplitude exceeding ±100 μV and the responses to the first two standard stimuli immediately after a deviant were rejected. The data from participants with less than 50 accepted epochs for any stimulus type were excluded from further analysis. The average number of remaining epochs per participant was 360 for the standard stimulus and 69 for the deviant stimulus.

The epochs were binned and averaged according to the stimulus type. ERP difference responses for each deviant type were calculated by subtracting the standard waveform from that of the deviant. Mean amplitudes of frontocentral channels (F3, Fz, F4, C3, Cz, and C4) were extracted for four 60 ms time windows based on the peak latencies observed in the grand-average deviant-minus-standard difference waveforms: 120–180 ms from stimulus onset (Time Window 1), 460–520 ms (Time Window 2), 520–580 ms (Time Window 3), and 620–680 ms (Time Window 4). Frontocentral channels were included in line with previous infant MMR studies (e.g., Choudhury and Benasich, 2011; Ylinen et al., 2017); this is also in line with the frontocentral dominance of the MMN in adults (Näätänen, 2001).

The presence of MMRs or prediction error responses in each condition and time window was tested using one-sample, two-tailed *t*-tests. This involved comparing the response amplitudes derived from deviant-minus-standard difference waveforms, averaged across frontocentral channels (F3, Fz, F4, C3, Cz, and C4), to zero (the baseline). Effect sizes were estimated using Cohen’s *d.* In addition, to compare the response amplitudes for the three deviant types within each time window, the amplitudes derived from deviant-minus-standard difference waveforms were submitted to one-way analyses of variance (ANOVA) with the factor *Deviant* (CD vs. AD vs. AX). The effect sizes are reported using the η^2^ measure. *Post-hoc* tests were conducted using Bonferroni-corrected t-tests (effect sizes: Cohen’s *d*).

## Results

**Figure 2:**
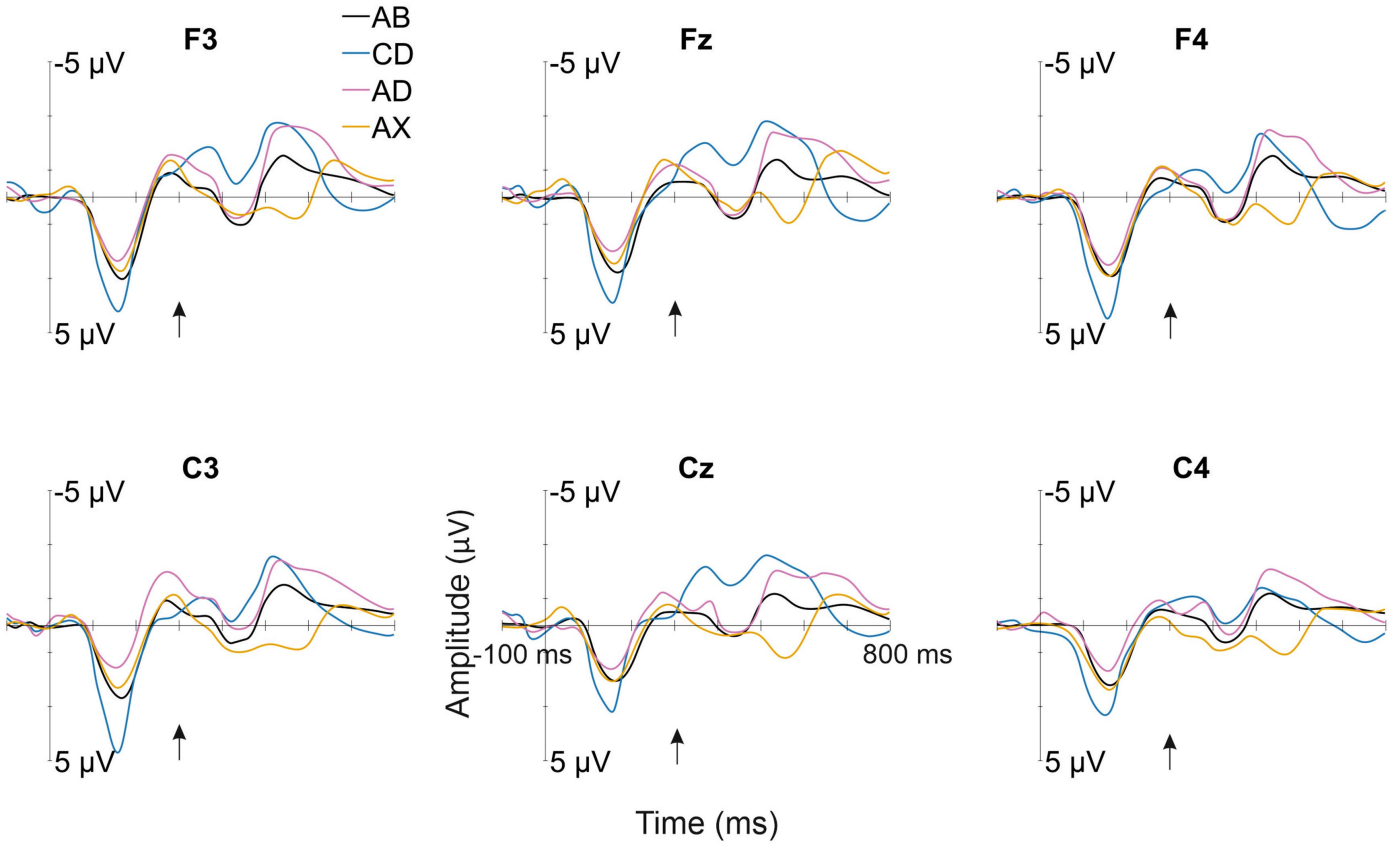
Group-averaged (N = 52) responses to the familiarized standard (AB—/kut:o/; black line) and the deviants (CD—/tek:a/, the other familiarized pseudoword; AD—/kuk:a/, the combination of the syllables of the two familiarized pseudowords forming a common word that infants might know; AX—/kup:e/, a novel pseudoword starting as the standard, but containing an unfamiliar syllable) from the electrode sites used in the statistical analyses. The Y-axis is at the stimulus onset, and the onset of the second syllable is marked with a black arrow.

**Figure 3:**
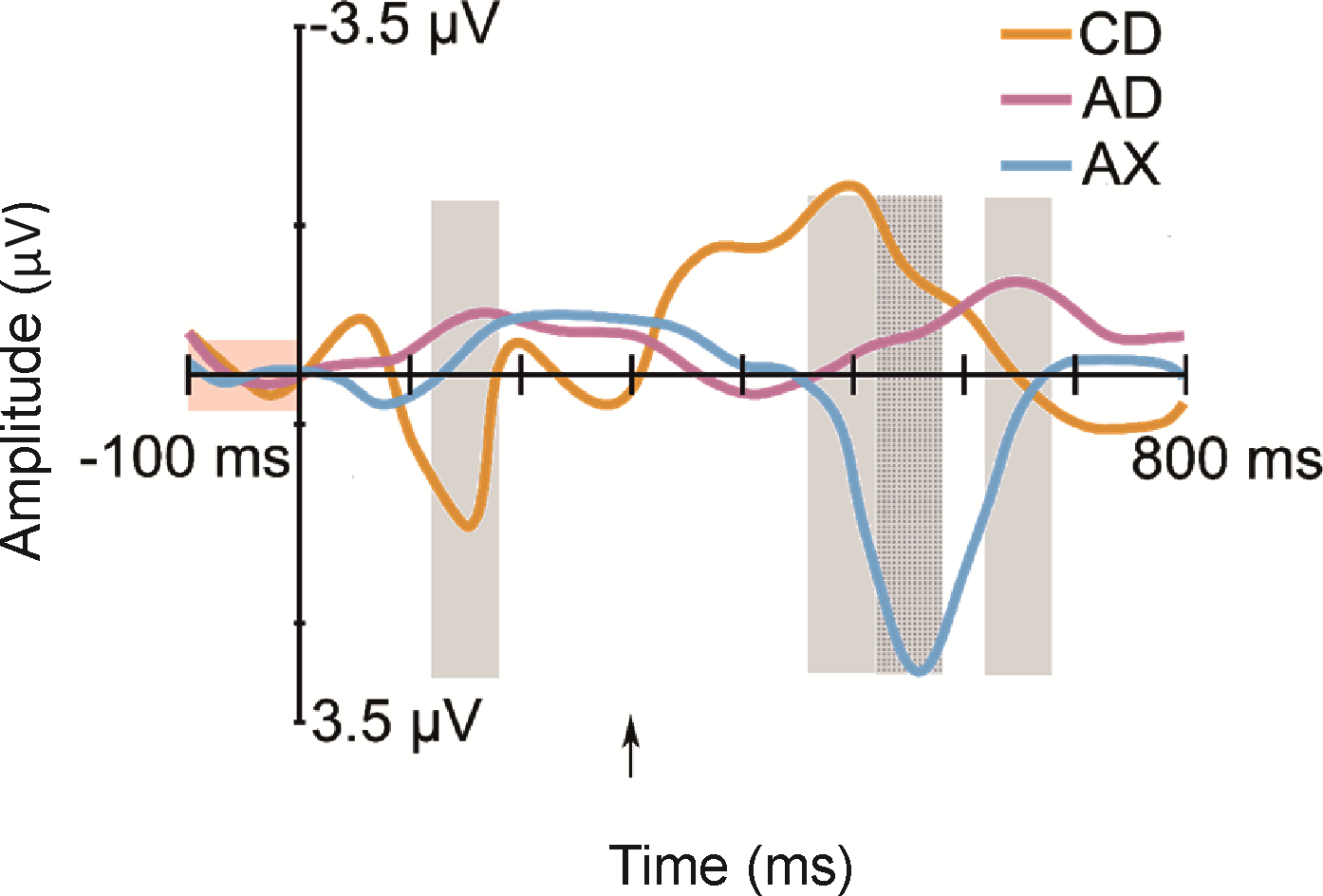
Group-averaged (N = 52) frontal (Fz) difference responses to the deviants: CD—/tek:a/, the other familiarized pseudoword besides the standard; AD—/kuk:a/, the combination of the syllables of the two familiarized pseudowords that forms a real word; AX—/kup:e/, a novel pseudoword starting as the standard, but containing an unfamiliar syllable. Measurement time windows are marked with light gray rectangles, and the baseline is marked with a light red rectangle. The Y-axis is at the stimulus onset, and the onset of the second syllable is marked with a black arrow.

All deviant types elicited a response that differed significantly from the baseline (see Table 1 for mean amplitudes derived from the deviant-minus-standard difference waveforms and *t*-test results for the significant responses in each time window; see Figure 2 for the original responses and Figure 3 for the group-averaged deviant-minus-standard waveforms).

**Table 1:** Deviant-minus-standard difference amplitudes significantly differing from zero and the results of the one-sample *t*-tests (two-tailed), separately for each deviant in each Time Window.

Difference response	CD /tek:a/ (vs. AB /kut:o/)	AD /kuk:a/ (vs. AB /kut:o/)	AX /kup:e/ (vs. AB /kut:o/)
Time Window 1 (120–180 ms)	**1.3 (2.9) *t*(51) = 3.2, *p* < 0.01, *d* = 0.44		
Time Window 2 (460–520 ms)	*−1.06 (3.3) *t*(51) = −2.3, *p* < 0.05, *d* = −0.32		
Time Window 3 (520–580 ms)	*−0.86 (3.1) *t*(51) = −2.01, *p* < 0.05, *d* = −0.28	*−0.70 (2.4) *t*(51) = −2.1, *p* < 0.05, *d* = −0.29	**2.07 (2.5) *t*(51) = 5.9, *p* < 0.001, *d* = 0.81
Time Window 4 (620–680 ms)		**−0.85 (2.3) *t*(51) = −2.7, *p* < 0.05, *d* = −0.37	

Mean amplitudes (in bold) and standard deviations (in parentheses) are given in μV for the average of the frontocentral channels. The *t*-statistics (*t*, df—degrees of freedom, in parentheses, *p*—significance level, Cohen’s *d*—effect size) are also shown. Statistical significance is marked with asterisks (*p < 0.05, **p < 0.01).

The ANOVA for Time Window 1 (120–180 ms, the 1st syllable) yielded a significant effect of *Deviant* [*F*(2,102) = 8.83, *p* < 0.001, η^2^ = 0.15]. Investigating this effect further with Bonferroni-corrected pairwise comparisons, the response to CD was significantly more positive than that to AD and AX [*p* < 0.01 for both, *d* = 0.49 and *d* = 0.57, respectively]. Similarly, the ANOVA for Time Window 2 (460–520 ms) showed a significant *Deviant* effect [*F*(2, 102) = 5.4, *p* < 0.01, η^2^ = 0.10], and Bonferroni-corrected pairwise comparisons showed that the response to CD was significantly more negative than that to AX [*p* < 0.01, *d* = 0.49]. In Time Window 3 (520–580 ms), the ANOVA also showed a significant effect of *Deviant* [F(2, 102) = 22.3, *p* < 0.001, η^2^ = 0.31]. Bonferroni-corrected pairwise comparisons revealed that the response elicited by AX was significantly more positive than those elicited by CD and AD [*p* < 0.001 for both, *d* = 0.82 and *d* = 0.87, respectively]. Furthermore, the *Deviant* effect was significant in the ANOVA for Time Window 4 (620–680 ms) [F(2, 102) = 4.33, *p* < 0.05, η^2^ = 0.08]. According to Bonferroni-corrected pairwise comparisons, AD elicited a significantly more negative response than either CD or AX [*p* < 0.05 and *d* = 0.37 for both].

## Discussion

The current study examined whether a newly learned word form elicits brain responses reflecting word-form recognition in 12-month-old infants or whether predictive processing is enabled by a learned rule. The answer to this question was assessed by measuring ERP responses to familiarized and unfamiliarized (pseudo)words. ERPs showed positive-polarity responses for the first and second syllable changes in pseudowords (CD and AX, respectively). However, the second syllables of a common word AD and a familiarized pseudoword CD elicited negative-polarity responses.

Confirming Hypothesis I (sequential deviation), all sequential deviants elicited responses that differed from the standard after their onset of deviation (Table 1 and Figure 3; Kushnerenko et al., 2013). The positive-polarity response to CD in Time Window 1 likely reflects MMR to the acoustic deviance of the first syllable of CD from the standard AB. In line with Hypotheses I (sequential deviation), II (within-word prediction error), and III (novelty response), the robust positive-polarity response in Time Window 3 elicited by the novel syllable X completing an unfamiliar word form likely reflects the sum of the word-level prediction error response (Ylinen et al., 2017; Suppanen et al., 2022), the novelty response (Kushnerenko et al., 2013), and the response to rare acoustic parameters (Kushnerenko et al., 2007). This interpretation of the functional distinction between the positive MMR to acoustic deviation for the first syllable of CD in Time Window 1 and the positive response to AX in Time Window 3 is also supported by different latencies from change onset (120–180 ms from the 1st syllable onset vs. 220–280 ms from the 2nd syllable onset).

Because sequential acoustic deviance resulted in positive-polarity responses in the current data (see also Kushnerenko et al., 2007), acoustic deviance cannot account for the negative response for the second syllable of AD, which differed significantly from the baseline in Time Window 4. In contrast, the observed response is in line with our earlier study (Ylinen et al., 2017), in which we found, in 12-month-old infants, a negative-polarity response for the syllable completing the word /kuk:a/, but not for the acoustically identical syllable /ka/ presented in isolation, and, thus, the observed negative-polarity response was explained by the activation of the word representation for /kuk:a/ (‘flower’). Similarly, in line with Hypothesis IV, the negative-polarity response elicited by the same word as in our previous study (referred to as AD in the current description; the only actual word delivered in the sequences) can be interpreted as reflecting word recognition via the activation of a word representation in the infant brain (see also Garagnani and Pulvermüller, 2011).

If the processing of newly learned word forms is dominated by predictive processing resulting from rule learning (Hypothesis V), then the response to the D syllable in the CD pseudoword should be suppressed because the infants have learned during familiarization that C is followed by D and, thus, C predicts D. The alternative Hypothesis VI, in turn, states that if the processing of newly learned word forms in the infant’s brain is dominated by recognition of the newly learned word form, then the D syllable in the CD pseudoword should elicit an enhanced response resembling that observed for AD (the actual word that could be known by the infants; see above). The pattern of current responses is compatible with the latter hypothesis: CD elicited a prominent negative-polarity response similar to AD rather than a suppressed response. Therefore, we interpret the negative-polarity response to the familiarized CD pseudoword as reflecting the activation of a newly established representation and the recognition of the word form for CD learned from speech exposure (during the familiarization phase). Thus, the present pattern of data supports Hypothesis VI, suggesting that successful word-level prediction (the first syllable predicting the second syllable) can be indexed by an enhanced ERP response of negative polarity at 12 months, even for newly learned word forms that had no long-term memory representation before.

Despite both AD and CD showing negative-polarity responses to D, there were also differences between these responses: the response to the common word AD peaked at a longer latency than that to CD, which may be explained by the recent familiarization (possibly higher activation state) of the pseudoword CD. In addition, the response to the common word AD was not as distinct as the one to the familiarized pseudoword, likely because, according to parental reports, not all infants did yet know the word /kuk:a/ (here, AD), which could cause variation in the individual responses and result in a less sharp or lower-amplitude response. (Note that in the study by Ylinen et al., 2017, the infants were familiarized with the word *kukka* beforehand, whereas in the current study, they were not.)

The current study has, however, some limitations. Using stimulus types that violate the infants’ expectations in different ways would have allowed us to obtain a more detailed picture of infants’ predictive inference. However, the experiment would probably have been too long for the infants. In addition, a control condition in which the same syllables of interest (here, the final syllables) were presented in isolation, without a word context, would have allowed us to tease apart factors that might contribute to infants’ responses, including the acoustic properties of the speech stimuli and the effect of word context in creating expectations about the future input. Again, such a control condition was not possible due to time constraints. The latter limitation concerns mostly the novel deviant AX; however, since the other two deviants, namely a potentially familiar word AD and a novel (pseudo)word CD, shared the same critical syllable D, the observed differences in the responses to their second syllable could not be explained by the acoustic properties of the stimuli.

In conclusion, in 12-month-old infants, a newly learned word form appears to elicit an ERP response of negative polarity, potentially reflecting word-form recognition and resembling the responses elicited by familiar words established in long-term memory. In contrast, acoustic changes and other prediction errors in a sequence consisting of (pseudo)words elicit ERP responses of positive polarity. This suggests that although predictive processing takes place, successful learning, which enables correct prediction, does not result in suppressed responses (*cf.* Heilbron and Chait, 2018).

## Data availability statement

The raw data supporting the conclusions of this article will be made available by the authors on request, without undue reservation.

## Ethics statement

The studies involving humans were approved by the Ethics Committee for Gynecology and Obstetrics, Pediatrics, and Psychiatry of the Hospital District of Helsinki and Uusimaa. The studies were conducted in accordance with local legislation and institutional requirements. Written informed consent for participation in this study was provided by the participants’ legal guardians/next of kin.

## Author contributions

SY: Conceptualization, Funding acquisition, Investigation, Methodology, Project administration, Resources, Supervision, Writing – original draft, Writing – review & editing. ES: Data curation, Formal analysis, Funding acquisition, Investigation, Visualization, Writing – original draft, Writing – review & editing. IW: Conceptualization, Writing – original draft, Writing – review & editing. TK: Conceptualization, Writing – original draft, Writing – review & editing.

## Footnotes

^1^The two voices in the learning phase were designed to address the infants’ processing of the voice; they will be reported separately. In the current study, the ERPs of the test phase were analyzed.
