# Supplementary material for: Establishing neural representations for new word forms in 12-month-old infants
Source: Front Hum Neurosci. 2024 Jun 13;18:1386207. doi: 10.3389/fnhum.2024.1386207 (PMC11208488; doi:10.3389/fnhum.2024.1386207)
Supplement: Supplementary file 1 [file Table_1.docx]

Supplementary materials

**Supplementary Table 1.** Stimulus properties (intensity, F0, and duration) in each speaker and syllable as measured with Praat (Boersma & Weenink, 2010). The two numbers in each cell denote two exemplars used. The first syllables are associated with the time window 1 and the 2^nd^ syllables with time windows 2-4 in the analysis used.

|  | Male speaker | | | | | Female speaker | | | | |
| --- | --- | --- | --- | --- | --- | --- | --- | --- | --- | --- |
|  | ku (1st syllable | te (1st syllable | ka (2nd syllable) | to (2nd syllable) | pe (2nd syllable) | ku (1st syllable | te (1st syllable | ka (2nd syllable) | to (2nd syllable) | pe (2nd syllable) |
| intensity (dB) | 80, 80 | 80, 80 | 81, 80 | 80, 80 | 80, 80 | 80, 80 | 80, 80 | 75, 75 | 75, 75 | 75, 75 |
| F0 (Hz) | 127, 126 | 122, 127 | 101, 98 | 100, 101 | 101, 101 | 226, 227 | 222, 228 | 187, 181 | 188, 181 | 187, 184 |
| duration (ms) | 91, 91 | 90, 90 | 126, 126 | 126, 126 | 126 126 | 90, 90 | 90, 90 | 127, 127 | 126, 126 | 127, 126 |
